# Effect of a multidisciplinary lifestyle intervention on stress-related parameters in people with rheumatoid arthritis and osteoarthritis: secondary analysis of the "Plants for Joints" randomized controlled trial

**DOI:** 10.1016/j.cpnec.2025.100298

**Published:** 2025-05-02

**Authors:** C.A. Wagenaar, J. Christiaans, V. Hermans, W. Walrabenstein, F.A. Koopman, H. van Middendorp, D. van Schaardenburg

**Affiliations:** a Reade Center for Rheumatology and Rehabilitation, Amsterdam, the Netherlands; b Department of Clinical Immunology and Rheumatology, Amsterdam University Medical Centers, University of Amsterdam, Amsterdam, the Netherlands; c Amsterdam Rheumatology and Immunology Center, Amsterdam, the Netherlands; d Institute of Psychology, Health, Medical, & Neuropsychology unit, Leiden University, Leiden, the Netherlands

**Keywords:** Rheumatoid arthritis, Osteoarthritis, Multidisciplinary lifestyle intervention, Stress, Secondary analysis

## Abstract

**Objective:**

In two randomized controlled trials, the Plants for Joints (PFJ) multidisciplinary lifestyle intervention reduced signs and symptoms of rheumatoid arthritis (RA), or metabolic syndrome-associated hip or knee osteoarthritis (MSOA) compared with usual care. This secondary analysis aimed to evaluate the effect of the PFJ intervention on stress-related parameters.

**Methods:**

In two PFJ observer-blind randomized controlled trials (RCT), people with (1) RA with low-moderate disease activity or (2) MSOA were randomized to receive the PFJ lifestyle intervention or usual care. The 16-week PFJ intervention consisted of a whole-food plant-based diet, physical activity, and sleep and stress management. This secondary analysis investigated stress-related outcomes including heart rate, heart rate variability (HRV: root square mean of successive differences (RMSSD) and normalised high frequency (HFnorm), salivary cortisol, perceived stress (Perceived Stress Scale 10; PSS-10), and time spent on stress-reducing activities. An intention-to-treat analysis with a linear regression model (HRV) or linear mixed model (cortisol and PSS-10), adjusted for baseline values, was used to analyse between-group differences.

**Results:**

77 people with RA and 64 with MSOA completed the RCT. RA participants following the PFJ intervention showed a significant improvement in HFnorm (between-group difference: 6.6; 95 % CI 0.5, 12.6) and an improving trend in RMSSD (4.3; 95 % CI -1.5, 10.1) alongside non-significant reductions of heart rate (3.1; 95 % CI-3.9, 10.1), salivary cortisol (1.3; 95 % CI -0.6, 3.1) and perceived stress (−2.0; 95 % CI -4.4, 0.3), compared to usual care. In participants with MSOA, there were no differences in heart rate, HRV outcomes, cortisol, or perceived stress between the intervention and control group. Both RA and MSOA participants temporarily increased time spent on stress-reducing activities, yet no change from baseline was observed after 16 weeks. In RA participants increased time spent on stress-reducing activities was associated with increased HFnorm.

**Conclusion:**

This secondary analysis suggests the PFJ program may have a positive influence on stress-related parameters in people with RA, but not MSOA, compared to usual care.

## Highlights

• Secondary analysis of two randomized controlled trials on stress-related parameters.
• Plants for Joints lifestyle intervention in rheumatoid arthritis and osteoarthritis.
• Outcomes include heart-rate variability, salivary cortisol and perceived stress.
• For the rheumatoid arthritis group significant improvement in HFnorm was found.
• In osteoarthritis no differences were found between intervention and control group.

## 1 Introduction

Rheumatoid arthritis (RA) is a chronic systemic inflammatory auto-immune disease which primarily affects the joints. RA has a prevalence of over 269.000 people in the Netherlands [1]. Osteoarthritis (OA) is a chronic degenerative joint disease, most commonly affecting spine or synovial joints such as knees and hips [2]. OA is the most prevalent form of arthritis in the Netherlands, with a prevalence of over 1,5 million people [3]. Metabolic syndrome-associated osteoarthritis (MSOA) is a distinct phenotype of OA, based on studies showing associations between OA and the components of metabolic syndrome [4]. Stress and other lifestyle components, including physical inactivity, obesity, poor diet, and disturbed sleep, contribute to systemic chronic low-grade inflammation which is a common driver of both RA and MSOA [5].

Stress refers to a process in which environmental demands exceed a person's adaptive capacity, leading to psychological, behavioural, and biological responses that may increase disease risk [6]. Chronic conditions like RA and MSOA significantly impact quality of life and may lead to greater stress due to chronic pain, stiffness, fatigue, long-term medication use, an unpredictable disease course, and its impact on an individual's work [7,8]. An altered immune function in people with RA, also results in a greater inflammatory cytokine and C-reactive protein (CRP) production compared to controls as a response to experimental stress [9]. Conversely, stress-related biopsychosocial risk factors (e.g. daily stress, pain catastrophizing and avoidance) and resilience factors (e.g. optimism and social support) influence the quality of life, physical symptoms of pain and fatigue, and inflammatory markers in patients with RA [7]. Furthermore, chronic stress, sleep deprivation, intake of high glycemic index foods, pain, and inflammation stimulate cortisol production, impacting body composition and metabolic health due to its association with higher body weight and waist circumference [10].

One of the major pathways activated by acute and chronic stress is the autonomic nervous system (ANS), consisting of the sympathetic (SNS) and the parasympathetic nervous system (PNS)[11]. The SNS and PNS are highly coordinated to maintain physiological homeostasis whereby the SNS responds to stress, and the PNS restores balance once the stress subsides [11]. However, prolonged stress keeps the SNS active, disrupting this balance. Heart Rate Variability (HRV) is a measure of the variation in time between consecutive heartbeats and provides insight into the ANS, particularly the balance between the PNS and SNS (1996). Neurobiological evidence links low HRV to high stress and supports its use for the evaluation of psychological health and stress [11], whereas a higher HRV indicates heightened parasympathetic control and is considered indicative of psychological resilience.

Studies examining various interventions in people with RA have found that stress management training and mindfulness-based interventions improved psychological functioning and general wellbeing, and reduced depressive symptoms, pain, psychological distress levels of anxiety, and cortisol levels [[12], [13], [14]]. While these interventions did not significantly affect the Disease Activity Score of 28 joints (DAS28), a review on the effect of meditation, mindfulness, and yoga in people with RA found multiple yoga-based intervention studies resulted in a decreased DAS28, number of inflamed joints, and systemic inflammatory markers (C-reactive protein (CRP) and erythrocyte sedimentation rate (ESR)) compared to controls [15]. Yoga has also been shown to have a positive effect on heart rate, blood pressure, HRV, and cortisol levels in people with RA in some studies, but not others [15,16]. Furthermore, a recent meta-analysis evaluating the effect of mindfulness-based interventions on immunity-related biomarkers showed a post-intervention reduction in CRP and IL-6 in people with various diagnoses including RA [17]. Electrical vagus nerve stimulation has also been shown to inhibit cytokine production and decrease disease activity [18]. The vagus nerve can also be stimulated by deep breathing exercises, resulting in an activation of the PNS as measured by HRV [19].

While only a few studies have evaluated stress-management techniques in people with OA, one study showed significant improvements in pain and function after a meditation program [20]. In another study, knee osteoarthritis patients with higher baseline ‘mindfulness’ scores were more likely to respond to exercise than patients with lower baseline mindfulness [21], suggesting possible synergistic effects between stress management and exercise therapy. Overall, these findings suggest interventions addressing stress may help attenuate inflammation and can contribute to improved health outcomes.

Considering the potential synergistic effects of combining lifestyle interventions, the Plants for Joints (PFJ) randomized controlled trial (RCT) investigated the effect of a multidisciplinary lifestyle intervention based on a whole food plant-based diet, physical activity, and sleep and stress management in people with low to moderately active RA or hip and/or knee MSOA [22]. After the 4-month intervention, disease activity was significantly decreased in people with RA (mean DAS28 –0.9 point), while those with MSOA had significantly less pain and stiffness and improved physical function (mean Western Ontario and McMaster Universities Osteoarthritis Index (WOMAC) score −11 points) compared with a usual care control group [23,23]. Both RA and MSOA groups had improved metabolic outcomes, including weight, fat mass, waist circumference, haemoglobin A1c (HbA1c) and low-density lipoprotein (LDL) cholesterol [23,23]. CRP levels decreased in both RA and OA intervention groups at the end of the RCT, but the reduction was only significant in the OA group [23,23]. Given the effect of stress-management interventions on inflammation and disease outcomes in previous research, it is hypothesized that the stress-component of the PFJ intervention contributed to its clinical effectiveness. Therefore, in this secondary analysis, the aim was to investigate whether the PFJ lifestyle intervention affected stress-related parameters in people with RA or MSOA, compared to usual care.

## 2 Methods

### 2.1 Design and study sample

Two assessor-blind open-label RCTs compared the effect of a multidisciplinary lifestyle intervention to usual care in people with (1) RA or (2) MSOA between May 2019 and December 2021 at the Reade rehabilitation and rheumatology clinic in Amsterdam, the Netherlands [22,23,23]. During the RCT, visits took place at baseline, 2 and 4 months. Study protocols were prospectively registered (International Clinical Trial Registry Platform numbers NL7800 and NL7801) and published [22].

People aged ≥18 years were included in the RCTs if they had (1) RA according to the ACR/EULAR 2010 criteria, with a low to moderate disease activity (2.6≤ DAS28 ≤ 5.1) and had stable treatment with or without disease-modifying antirheumatic drugs (DMARDs) for at least 3 months [25,26] or (2) hip and/or knee OA according to the ACR clinical criteria and metabolic syndrome according to the National Cholesterol Education Programme criteria [[27], [28], [29]]. 89 % of participants fulfilled ACR radiological criteria for OA, 47 (knee, 73 %) and 50 (hip, 78 %) participants had a Kellgren-Lawrence grade between 2 and 4 [23,24,28,30]. People with a body mass index <18.5kg/m2, already following a plant-based diet, unwilling to quit smoking, and pregnant women were excluded. Participants were asked to not make medication changes during the study, changes were reported as protocol deviations. In this secondary analysis, results are shown separately for RA and MSOA but are combined in one report as the same intervention was used.

### 2.2 Intervention

Details of the PFJ intervention were previously published [22,23,23]. Briefly, participants received individual intakes with a registered dietitian and a physical therapist at the start of the intervention. During the 4-month intervention mixed groups of RA and MSOA, participants received theoretical and practical education about a whole food plant-based diet, physical activity, and sleep and stress management during 10 group meetings of 6–12 participants [31,32]. This included a calorie-unrestricted plant-based version of the Guidelines on Healthy Nutrition from the Health Council of the Netherlands [31], personal physical activity goals in accordance with the Dutch physical activity guidelines (150 min/week moderate intense physical activity and 2 days/week musculoskeletal strengthening activities) [32], psychoeducation on the effects of psychological stress on health and stress management and coaching on sleep. Stress management was based on protocols by De Brouwer et al. [13]. Subjects received psychoeducation on the effects of stress on health and on stress management, as well as guided practice and home exercises (supported by tools incl. audio recordings and apps) on relaxation techniques and breathing and visualization exercises. Education was provided by registered dietitians, a physical therapist, personal trainers, and therapists with expertise in sleep and stress reduction. During the intervention, participants were facilitated with general information and videos, exercises for at home, fully elaborated weekly menus and daily supplementation with methylcobalamin (1500 μg) and cholecalciferol (50 μg).

### 2.3 Outcomes

General anthropometric measurements, including BMI, waist circumference, and blood pressure were measured and previously published [23,23]. Stress-related parameters evaluated in this study were heart rate, heart rate variability (HRV), salivary cortisol, and perceived stress level. Number of stress-reducing activities were self-reported using a digital questionnaire. Other lifestyle components including dietary intake and physical activity were also measured to assess adherence to the intervention. “MijnEetmeter” a validated digital food diary [33] was used to measure dietary intake. Participants were asked to complete this diary at least 4 days out of every week. A 2-day dietary recall was performed for participants having difficulty with filling in the food diary themselves. Minutes of physical activities in the past week were assessed with a digital questionnaire.

#### 2.3.1 Heart rate and heart rate variability

Heart rate and HRV were measured in a supine position with a 5-min electrocardiography (ECG) using the SphygmoCor EM3, at baseline and at 16 weeks. For this analysis RMSSD and HFnorm were chosen because they both represent parasympathetic nervous system activity. Although they are highly correlated with each other [34,35], both were included to enable comparison with the existing literature. An increase in RMSSD and HFnorm reflects more parasympathetic activity, indicating a lowered stress state.

#### 2.3.2 Cortisol

At baseline, week 8, and the end of the 16-week program, fasted salivary cortisol was collected by participants themselves using an at-home collection kit. Salivary cortisol was chosen due to its collection and processing advantages (e.g. non-invasive, at-home collection, sample stability, and lower costs) over serum cortisol [36], and its high correlation with serum cortisol as a measure of free cortisol [36,37]. Salivary cortisol is therefore a feasible and effective measurement tool for stress research [38]. Participants were instructed to collect the samples within 30 min of waking up and before brushing their teeth. Participants were asked to freeze the sample at home and transport it to Reade for their next visit, where it was stored at −80 °C. Salivary cortisol (nmol/l) was analyzed using the liquid chromatography–tandem mass spectrometry (LC-MS/MS) method at the Endocrinology Laboratory of the Amsterdam UMC.

#### 2.3.3 Perceived Stress Scale

A Dutch version of the Perceived Stress Scale (PSS-10) was used as a subjective measure of stress. The PSS-10 is a 10-item survey which measures perceived stress over the past month [39]. It is frequently used and has excellent psychometric properties [40,41]. The questionnaire was filled out by participants online at baseline, week 8, and week 16. The score on the PSS-10 ranges from 0 to 40, with a score of zero meaning no perceived stress.

### 2.4 Statistical analysis

All analyses were conducted for RA and MSOA separately. Heart rate and HRV measures (two time points) were analyzed with linear regression adjusting for baseline values of the particular outcome. Cortisol and Perceived Stress Scale data (three time points) were analyzed using linear mixed effects models, with random effects for subjects and fixed effects for group and baseline values of the particular outcome. An additional subgroup cortisol analysis was performed excluding people using systemic and inhalation glucocorticoids or hormone-replacement therapy. Additional analyses were conducted whereby the models were adjusted for potential confounders including sex, age, and BMI at baseline. For stress-related outcomes that were significantly different in the intervention group as compared to the control group at the end of the study, correlations with disease-related outcomes (e.g. DAS28 for RA) were assessed using Pearson's (normally distributed data) or Spearman's (skewed data) correlation coefficients. Correlations between cortisol and waist circumference were also assessed. Multiple linear regression analyses were also performed to explore associations between changes in adherence to the lifestyle components (stress-reducing activities (min/week), physical activity (min/week), fiber intake (g/1000 kcal), saturated fat intake (energy%)), and changes in stress-related outcomes in the intervention groups from start to end of the intervention. Outliers were manually evaluated and removed if they showed a high probability of measurement error. All analyses followed an intention-to-treat approach with significance set at P < 0.05, conducted using IBM SPSS Statistics version 29. Number of stress-reducing activities were analyzed using descriptive statistics.

## 3 Results

### 3.1 Baseline characteristics

In the RA group, 77 people (93 %) completed the RCT and were analyzed [23,24]. Study participants had a mean age of 55 years, were mostly female (92 %), had a mean BMI of 26 kg/m^2^, a mean disease duration of 8.8 years, and a mean DAS28 of 3.85. In the MSOA group, 64 people (97 %) completed the RCT and were analyzed [23,24]. Study participants had a mean age of 64 years, were mostly female (84 %), had a mean BMI of 33 kg/m^2^, and a mean WOMAC score of 39.

Four HRV and three cortisol measurements were removed due to a high probability of measurement error (HRV *n* = 1 heart rate of 35/min, *n* = 4 inconclusive measurements; cortisol *n* = 3 three times higher than median (possible sample contamination with blood)). Baseline values of heart rate, HFnorm and PSS-10 were similar for RA and MSOA participants. Median baseline RMSSD values, however, were lower in the MSOA group, compared to the RA group (24.1 ms vs. 31.9 ms, respectively). The median baseline cortisol level of the MSOA group (8.1 nmol/L) was higher than the median cortisol level of the RA group (6.9 nmol/L). Table 1

**Table 1:** Baseline characteristics of Plants for Joints rheumatoid arthritis (RA) trial.

Characteristic	Plants for Joints group	Control group
n =	40	37
Age, years	56.4 (13.4)	52.8 (10.3)
Female sex, number (%)	36 (90 %)	35 (95 %)
Disease duration, years	6.5 (3.0–14.0)	5.0 (1.5–14.5)
DAS28	3.90 (0.69)	3.79 (0.71)
BMI, kg/m2	27.1 (4.6)	25.2 (3.7)
Heart rate, bpm	66 (7.9)	65 (6.7)
RMSSD, ms	23.6 (13.8–42.3)	37.1 (27.0–47.4)
HFnorm, n.u.	36.1 (19.6–64.7)	42.3 (25.2–58.7)
Cortisol, nmol/L	8.7 (3.5–12.0)	5.6 (2.7–10.6)
PSS-10	14.0 (6.4)	16.1 (4.7)

Data is shown as mean (SD) when normally distributed or as median (interquartile range) when skewed. DAS28 = 28-joint Disease Activity Score, n.u. = normalised units (calculated by dividing the component in question by the total power subtracted by the VLF (very low frequency) component), PSS-10 = Perceived Stress Scale 10-item questionnaire (score ranges from 0 to 40; 0 = no perceived stress).

### 3.2 Heart rate and heart rate variability

At baseline, heart rate Table 2 was comparable between the PFJ and control groups in both the RA and MSOA trials. Heart rate decreased in all study groups during the trials, leading to a non-significant difference between the intervention and control groups after 16 weeks (Table 3, Table 4).

**Table 2:** Baseline characteristics of Plants for Joints metabolic syndrome-associated osteoarthritis (MSOA) trial.

Characteristic	Plants for Joints	Control group
*n* =	32	32
Age, years	63.3 (6.8)	63.4 (6.1)
Female sex, number (%)	28 (88 %)	26 (81 %)
BMI, kg/m2	33.2 (5.2)	33.4 (5.7)
Heart rate, bpm	67 (62–72)	66 (60–73)
Location OA		
Hip OA, number (%)	7 (22 %)	5 (16 %)
Knee OA, number (%)	9 (28 %)	16 (50 %)
Hip and Knee OA, number (%)	16 (50 %)	11 (34 %)
WOMAC total score	38.5 (13.4)	40.1 (19.0)
RMSSD, ms	23.7 (14.9–38.8)	27.3 (16.8–42.8)
HFnorm, n.u.	40.7 (20.0)	38.4 (23.5)
Cortisol, nmol/L	8.4 (4.7)	9.2 (3.8)
PSS-10	11.9 (6.9)	13.7 (7.1)

Data is shown as mean (SD) when normally distributed or as median (interquartile range) when skewed. WOMAC = Western Ontario and McMasters Universities Osteoarthritis Index (range 0–96). n.u. = normalised units (calculated by dividing the component in question by the total power subtracted by the VLF (very low frequency) component), PSS-10 = Perceived Stress Scale 10-item questionnaire (score ranges from 0 to 40; 0 = no perceived stress).

**Table 3:** Stress-related parameters of Plants for Joints rheumatoid arthritis (RA) trial.

	Plants for Joints group			Control group			Between group difference (95 % CI)	*p*-value
	Baseline	8 weeks	16 weeks	Baseline	8 weeks	16 weeks		
Heart rate, bpm	n = 37		n = 37	n = 34		n = 35		
	66 (8)	–	61 (28.2)	65 (6.7)	–	57 (39.7)	3.1 (−3.9, 10.1)	0.4
RMSSD, ms	n = 37		n = 36	n = 34		n = 33		
	23.6 (13.8–42.3)	–	26.1 (12.5–44.9)	37.1 (27.0–47.4)	–	25.6∗ (17.5–34.3)	4.3 (−1.5, 10.1)	0.2
HFnorm, n.u.	n = 37		n = 36	n = 34		n = 33		
	36.1 (19.6–64.7)	–	39.3 (27.3–62.4)	42.3 (25.2–58.7)	–	31.7∗ (20.3–48.7)	6.6 (0.5, 12.6)	0.03
PSS-10	n = 30	n = 31	n = 36	n = 29	n = 33	n = 33		
	14.0 (6.4)	12.6 (6.3)	11.9 (6.3)	16.1 (4.7)	16.2 (6.0)	14.9 (6.1)	−2.0 (−4.4, 0.3)	0.09
Cortisol, nmol/L *all participants*	n = 36	n = 23	n = 22	n = 29	n = 25	n = 23		
	8.7 (3.5–12.0)	7.0 (3.5–10.1)	6.9 (4.1–12.6)	5.6 (2.7–10.6)	5.7 (2.8–8.7)	6.4 (3.0–8.3)	1.3 (−0.6, 3.1)	0.2
Cortisol, nmol/L *excluding corticosteroids*	n = 24	n = 16	n = 16	n = 17	n = 17	n = 16		
	9.6 (4.9–11.6)	7.1 (4.5–9.9)	7.4 (5.3–12.3)	6.5 (3.6–10.6)	7.1 (4.5–10.0)	7.0 (3.5–8.5)	0.6 (−1.6, 2.7)	0.6
Stress-reducing activities, min/week	n = 40	n = 40	n = 38	n = 32	n = 32	n = 35		
	20.0 (0.0–34.5)	32.5 (2.8–53.8)	21.0 (0.0–44.0)	33.0 (11.0–66.0)	33.0 (20.0–55.0)	35.0 (20.0–77.0)	–	–

Results shown as mean (SD) when normally distributed or as median (interquartile range) when skewed. P values indicate between group differences adjusted for baseline values at the end of the 16-week trial. ∗ = within group difference significant (p < 0.05), ms = milliseconds, n.u. = normalised units, PSS-10 = Perceived Stress Scale 10-item questionnaire (score ranges from 0 to 40; 0 = no perceived stress). After adjusting models for baseline age, sex, and BMI all outcomes were the same except for HFnorm which was not significant (6.3 95 % CI (−0.01, 12.6)).

**Table 4:** Stress-related parameters of Plants for Joints metabolic syndrome-associated osteoarthritis (MSOA) trial.

	Plants for Joints group			Control group			Between group difference (95 % CI)	*p*-value
	Baseline	8 weeks	16 weeks	Baseline	8 weeks	16 weeks		
Heart rate, bpm	n = 31		n = 31	n = 29		n = 28		
	67 (62–72)	–	59 (57–70)	66 (60–73)	–	49 (58–69)	3.0 (−10.7, 16.7)	0.7
RMSSD, ms	n = 31		n = 30	n = 29		n = 25		
	23.7 (14.9–38.8)	–	26.1 (16.0–35.6)	27.3 (16.8–42.8)	–	25.4 (17.9–37.8)	−0.4 (−6.9, 6.1)	0.9
HFnorm, n.u.	n = 31		n = 30	n = 29		n = 25		
	39.9 (23.6–58.9)	–	40.1 (26.4–52.6)	39.2 (19.0–54.9)	–	39.7 (23.3–58.2)	−0.8 (−7.7, 6.0)	0.8
PSS-10	n = 29	n = 29	n = 24	n = 27	n = 27	n = 28		
	11.0 (6.5–16.5)	10.0 (6.5–14.5)	9.5 (6.3–13.0)	12.0 (8.0–19.0)	12.0 (7.0–14.0)	11.0 (8.0–19.5)	0.3 (−2.0, 2.7)	0.8
Cortisol, nmol/L *all participants*	n = 30	n = 22	n = 21	n = 19	n = 17	n = 22		
	7.2 (5.1–10.8)	8.7 (3.6–10.3)	8.2 (5.1–12.0)	8.8 (7.3–12.2)	8.4 (5.9–13.9)	8.3 (5.3–10.3)	−1.4 (−3.6, 0.8)	0.2
Cortisol, nmol/L *excluding corticosteroids*	n = 24	n = 17	n = 18	n = 18	n = 15	n = 18		
	7.1 (5.2–11.4)	6.6 (3.5–9.6)	8.1 (5.3–12.5)	8.5 (7.0–10.9)	10.3 (6.2–15.0)	8.3 (5.9–9.6)	−1.2 (−3.5, 1.1)	0.3
Stress-reducing activities, min/week	n = 30	n = 29	n = 30	n = 32	n = 32	n = 31		
	26.0 (0.0–50.0)	40.0 (13.5–68.0)	26.0 (9.5–71.8)	33.0 (0.0–55.0)	44.0 (0.3–70.0)	33.0 (11.0–70.0)	–	–

Results shown as mean (SD) when normally distributed or as median (interquartile range) when skewed. P values indicate between group differences adjusted for baseline values at the end of the 16-week trial. ∗ = within group difference significant (p < 0.05), ms = milliseconds, n.u. = normalised units, PSS-10 = Perceived Stress Scale 10-item questionnaire (score ranges from 0 to 40; 0 = no perceived stress).

In the RA RCT, baseline values of RMSSD and HFnorm differed between the PFJ and control groups (23.6 ms vs. 37.1 ms and 36.1 n.u vs. 42.3 n.u., respectively) (Table 1). The PFJ group showed an increase in HFnorm from baseline to 16 weeks, while the HFnorm in the control group decreased resulting in a mean group difference of 6.6 (95 % CI 0.5, 12.6) (Table 3). The same trend was seen for RMSSD, although this was not statistically significant: mean group difference 4.3 (95 % CI -1.5, 10.1). In the MSOA trial, baseline values of RMSSD differed between the PFJ group and the control group (23.7 vs. 27.3 ms) (Table 2). No between-group differences in HRV measures were observed at 16 weeks (Table 4).

### 3.3 Cortisol

RA baseline cortisol levels were higher in the PFJ group compared to the control group (8.7 nmol/L vs. 5.6 nmol/L) (Table 1). The PFJ group showed a decrease over time, while levels in the control group increased slightly, with a non-significant mean group difference of 1.3 (95 % CI -0.6, 3.1) (Table 3). In a subgroup analysis, excluding patients using systemic and inhalation glucocorticoids (*n* = 14 PFJ, *n* = 13 control), a similar, non-significant trend was found (mean group difference 0.6 95 % CI -1.6, 2.7).

In MSOA participants, a difference in baseline mean cortisol values was also found between the PFJ and control group (8.4 nmol/L vs. 9.2 nmol/L) (Table 2), although less pronounced than in RA participants. From baseline to 16 weeks, cortisol levels increased in the PFJ group, while decreasing slightly in the control group (non-significant mean group difference −1.4 95 % CI -3.6, 0.8) (Table 4). In a subgroup analysis, excluding patients using systemic and inhalation glucocorticoids (*n* = 6 PFJ, *n* = 3 control) and hormone-replacement therapy (*n* = 1 PFJ, *n* = 1 control), a similar, non-significant trend was found (mean group difference −1.2 95 % CI -3.5, 1.1).

### 3.4 Perceived stress scale

In the RA trial, the PFJ intervention non-significantly reduced PSS-10 scores compared to the control group, with a mean group difference of −2.0 (95 % CI -4.4, 0.3) (Table 3). No differences between the PFJ group and the control group in PSS-10 scores were seen in the MSOA trial (Table 4).

### 3.5 Number of stress-reducing activities and program adherence

In both RA and MSOA RCTs there was an increase in stress-reducing activities in the PFJ group from baseline to 8 weeks (Table 3, Table 4). From 8 to 16 weeks, the number of stress-reducing activities decreased again to baseline levels. In the control groups, the number of stress-reducing activities was approximately the same between baseline and 16 weeks.

Adherence to the diet and physical activity recommendations were previously published [23,23]. Overall, RA and MSOA participants increased fiber and reduced saturated fat intake, supporting adherence to a whole-food plant-based diet. Physical activity was sufficient at baseline (recommendation >150 min/week) in both trial arms, increasing from 154 to 205 min/week in RA participants and remaining stable (±200 min *per*week) in MSOA participants.

### 3.6 Correlations and associations

No significant correlation was found between HFnorm and the change in DAS28. Also, there was no significant correlation found between waist circumference and cortisol for either study population. In the RA group, more time spent on stress-reducing activities was significantly associated with improvements in HFnorm (β = 0.38, SD = 30 min/week, *p* = 0.048), and increased physical activity was linked to higher perceived stress scores (β = 0.022, SD = 139 min/week, *p* = 0.025), though the effect was small. No significant associations were found between changes in fiber or saturated fat intake with stress outcomes. In the MSOA group, greater time spent on physical activity was associated with lower perceived stress scores (β = −0.025, SD = 119 min/week, *p* = 0.038), with no other significant associations observed for the other lifestyle outcomes.

## 4 Discussion

The 16-week ‘Plants for Joints’ lifestyle intervention significantly decreased disease activity in people with RA and improved pain, stiffness, and physical function in people with hip and/or knee MSOA [23,23]. This secondary analysis aimed to explore the intervention's impact on stress-related parameters to gain a greater understanding of whether the stress component of the intervention contributed to its clinical effectiveness. RA participants following the PFJ intervention showed a significant improvement in HFnorm and an improving trend in RMSSD alongside non-significant reductions of heart rate, salivary cortisol, and PSS-10 score, compared to usual care. In MSOA participants, no significant differences between the intervention and control group were found regarding heart rate, HRV measures, cortisol, or PSS-10 score. In both RA and MSOA intervention groups, participants initially increased time spent on stress-reducing activities, yet returned to baseline levels by the end of the RCTs. in RA participants, more time spent on stress-relieving activities was associated with greater improvements in HFnorm, while more time spent on physical activity was associated with a small, but significant increase in perceived stress. Conversely, in MSOA participants, more time spent on physical activity was associated with less perceived stress.

Compared to controls, the RA PFJ participants showed a significant improvement in HFnorm and an improving trend in RMSSD, indicating more parasympathetic activity, suggesting a reduced stress state. This is supported by two studies in people with RA, one comparing transcutaneous auricular vagus nerve stimulation to deep breathing, and the other on the effect of exercise, showing a significant improvement in RMSSD within intervention groups, or compared to sedentary controls, respectively [19,42]. T he disparity in RMSSD significance between PFJ and these studies may stem from differences in intervention types (at-home practice vs. guided activities). Furthermore, Ganesan et al. also showed a significant increase in HFnorm after yoga therapy compared to usual care in people with RA [16]. Contrastingly, for MSOA participants no difference was observed between the intervention and control groups in HRV-parameters. This is in line with a study in people with knee OA, which found no effect of a deep slow-breathing program on HRV parameters, compared to the control group [43]. To the best of our knowledge, this is the only study which evaluated the effect of an intervention on HRV parameters in people with OA.

In RA participants, the PFJ intervention led to a non-significant reduction of salivary cortisol levels, compared to usual care. This is in line with two studies showing stress-management training or yoga therapy reduced salivary cortisol response after a stress test or serum cortisol, respectively, in people with RA compared to controls [13,16]. In contrast with expectations, for MSOA patients, cortisol levels non-significantly increased in the PFJ group, compared to the control group. There are currently no other studies available which have assessed the effect of lifestyle interventions on cortisol levels in people with OA.

Both RA and MSOA intervention groups showed a trend towards an improvement in PSS-10 score, RA greater than MSOA, although there was no significant difference observed compared to controls at the end of the study. Other studies on stress-reducing techniques demonstrate varying results regarding perceived stress. A yoga intervention in sedentary adults with RA (*n* = 37) or knee OA (*n* = 38) showed a significant improvement of the PSS-10 score compared to baseline [44]. Contrastingly, other studies found no difference in PSS-10 score after a yoga or meditation intervention in people with RA or OA, respectively [20,45].

The differing results of the stress-related outcomes between RA and MSOA participants could be attributed to RA participants' potentially higher motivation to manage stress, given its established link to RA [7], or their baseline levels being either lower (HFnorm) or higher (PSS-10, cortisol) compared to MSOA, allowing for more potential improvement. However, the former was not reflected in the stress-reducing activity data.

In both RA and MSOA intervention groups, time spent on stress-reducing activities increased at week eight but returned to baseline by the end of the 16-week intervention. Trends towards improvements in HRV-outcomes, cortisol, and PSS-10 in the RA population suggest a potential stress-reducing effect of the PFJ intervention. It was hypothesized that the stress component of the PFJ intervention impacted stress outcomes and thus contributed to its clinical effectiveness. Although this was not reflected in the stress-reducing activity data, in the RA group there was significant association between stress-reducing activities and HFnorm, Additionally, participants may have become more mindful of stressors and employed stress-reduction techniques which were not quantifiable but could have contributed to the found trends. Furthermore, the short-term increase in stress-reducing activities may potentially have lasting longer-term effects. Alternatively, the intervention's overall impact on well-being, pain, physical function, activity, and self-management could have contributed to stress reduction [23,23,46,47]. Physical activity also appeared to influence perceived stress, with greater activity linked to higher perceived stress in RA participants and lower stress in MSOA participants. While the effects of individual lifestyle components cannot be isolated, the associations suggest that greater engagement in stress-reduction activities and physical activity were most linked to improvements in stress outcomes. However, these associations should be interpreted with caution, as adherence data was self-reported, the effects were small and potentially clinically insignificant, the sample size was limited, and baseline physical activity levels were already high.

### 4.1 Strengths and limitations

This secondary analysis exhibits strengths in its utilization of various stress-related parameters (subjective and objective) and inclusion of two patient populations (RA and MSOA) for comparison. However, the study's combined lifestyle intervention prevents the assessment of the impact of individual components, and its lack of power on stress-related parameters may have resulted in an inadequate sample size, hindering the ability to detect changes. To better explore the individual effects of diet, physical activity, sleep, and stress-reducing activities on clinical and stress-related outcomes, head-to-head comparisons with adequately powered sample sizes should be conducted. Furthermore, baseline value disparities between intervention and control groups in both trials may have influenced between-group change detection despite baseline value adjustments.

The method of cortisol measurement poses various limitations. Due to cortisol's circadian rhythm and pulsatile release [48], small differences in sample timing can lead to wide data distribution, reducing statistical power and potentially explaining non-significant results. Also, salivary cortisol, affected by acute stress and intra- and interindividual differences [50], is less reliable compared to hair cortisol, which provides a more accurate measure of long-term stress with fewer intra-individual differences [49]. Moreover, the presence of numerous missing values further decreases the analyses' power.

Finally, the participants received education on stress mechanisms and stress-reducing techniques, but there were no requirements as to the amount of time participants should engage in stress-reducing activities. A larger impact may have been observed if participants had been stimulated to do more (specific or guided) stress-reducing activities. Additionally, simultaneously addressing multiple lifestyle components in 16 weeks might be insufficient to induce significant change in each component, while addressing one component at a time consecutively could lead to greater effects. In future studies, a longer intervention period and strategies to enhance engagement with specific or guided stress-reducing activities, may better capture the effects of stress-reduction on clinical outcomes.

## 5 Conclusion

This secondary analysis of two randomized controlled trials on the effects of the Plants for Joints (PFJ) lifestyle intervention suggests a potential positive impact on stress-related parameters in people with RA, but not MSOA, compared to usual care. While participants only showed a short-term increase in observed time spent on stress-reducing activities, in RA participants increased time spent on these activities was associated with improved parasympathetic activity, measured by HFnorm. The intervention as a whole, other lifestyle components, or unquantified stress-related changes such as improved disease activity may have also contributed to reduced stress levels. These stress-related improvements may contribute to the observed clinical effectiveness of the PFJ intervention in people with RA.

## CRediT authorship contribution statement

**C.A. Wagenaar:** Writing – review & editing, Writing – original draft, Visualization, Validation, Methodology, Formal analysis, Data curation, Conceptualization. **J. Christiaans:** Writing – review & editing, Writing – original draft, Visualization, Validation, Methodology, Formal analysis, Conceptualization. **V. Hermans:** Writing – review & editing, Writing – original draft, Methodology, Formal analysis, Conceptualization. **W. Walrabenstein:** Writing – review & editing, Methodology, Data curation, Conceptualization. **F.A. Koopman:** Writing – review & editing, Conceptualization. **H. van Middendorp:** Writing – review & editing, Conceptualization. **D. van Schaardenburg:** Writing – review & editing, Supervision, Project administration, Conceptualization.

## Ethics approval

The PFJ RCT was approved by the Medical Ethical Committee of the Amsterdam University Medical Center, location VUmc with protocol identification number NL66649.048.18.

## Availability of data and materials

The datasets during and/or analyzed during the current study are available from the corresponding author on reasonable request.

## Declaration of generative AI and AI-assisted technologies in the writing process

During the preparation of this work the authors used ChatGPT in order to improve readability and language. After using this tool, the authors reviewed and edited the content as needed and take full responsibility for the content of the publication.

## Role of the funding source

The PFJ RCT was funded by Reade (Amsterdam, the Netherlands), Reade Foundation (Amsterdam, the Netherlands), Stichting Vermeer 14 (private foundation, Amsterdam, the Netherlands), and W.M. de Hoop Stichting (private foundation, Bussum, the Netherlands). The position of C.A.W. is funded by The 10.13039/501100001826Netherlands Organisation for Health Research and Development (ZonMw) no. 555003210. The funders had no role in the design and conduct of the study; collection, management, analysis, and interpretation of the data; preparation, review, or approval of the manuscript; and decision to submit the manuscript for publication.

## Declaration of competing interest

The authors declare the following financial interests/personal relationships which may be considered as potential competing interests: Authors **C.A.W., W.W., and D.v.S.** hold shares in Plants for Health, a limited liability company, which aims to have a positive impact on society and the environment and provide an adapted version of the Plants for Joints program as an additional treatment option for people with rheumatic conditions. All other authors report no conflict of interest.
